# Supplementary figures and images for: Efficient High-Throughput DNA Breathing Features Generation Using Jax-EPBD
Source: bioRxiv. 2024 Dec 12:2024.12.06.627191. Preprint. [Version 1] doi: 10.1101/2024.12.06.627191 (PMC11661089; doi:10.1101/2024.12.06.627191)

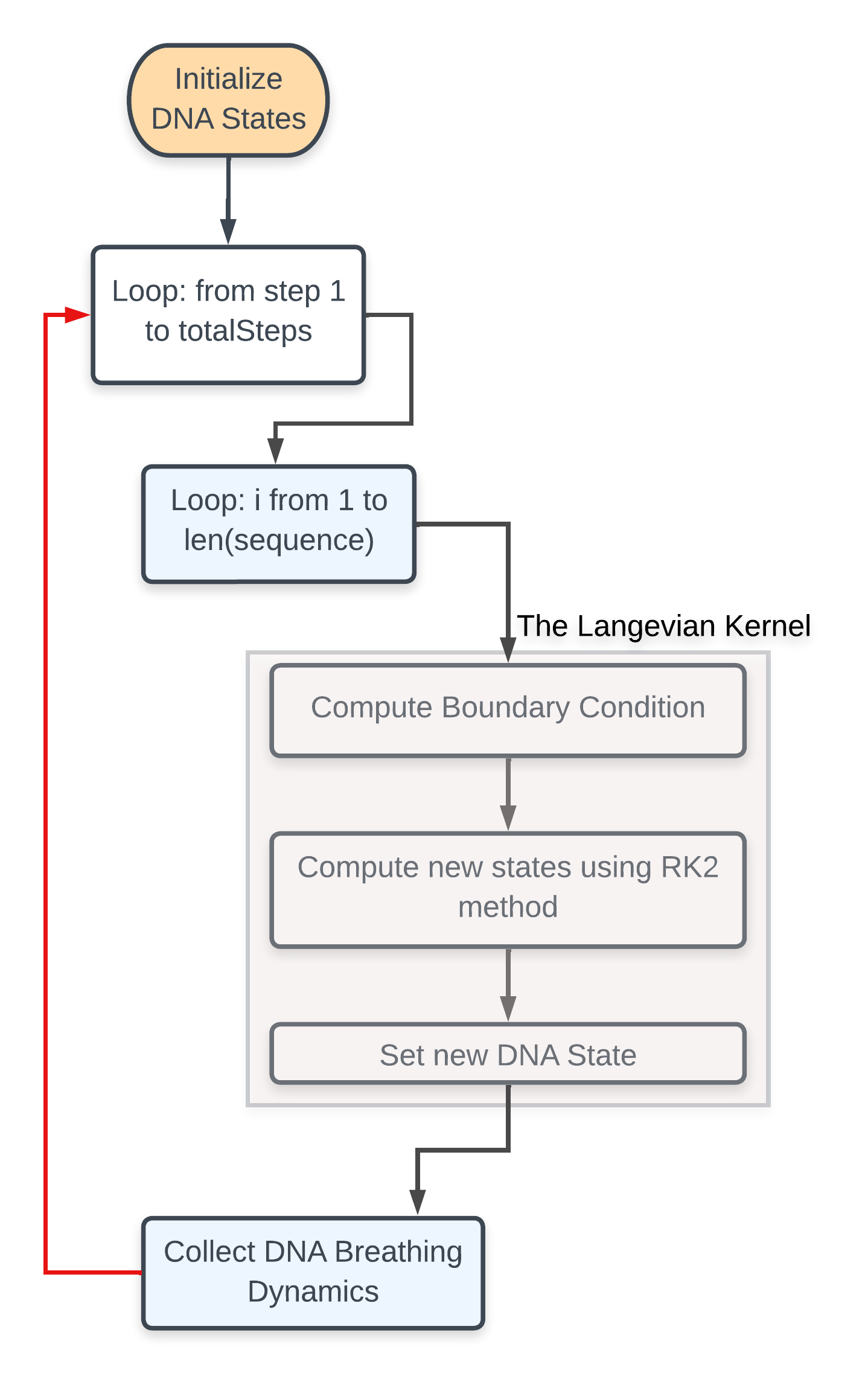

Supplement: Supplement 1 [file media-1.jpg]
